# Genotypic and Phenotypic Characterization of Fecal *Staphylococcus epidermidis* Isolates Suggests Plasticity to Adapt to Different Human Body Sites

**DOI:** 10.3389/fmicb.2020.00688

**Published:** 2020-04-21

**Authors:** Enriqueta Garcia-Gutierrez, Calum J. Walsh, Lizbeth Sayavedra, Teresa Diaz-Calvo, Dinesh Thapa, Patricia Ruas-Madiedo, Melinda J. Mayer, Paul D. Cotter, Arjan Narbad

**Affiliations:** ^1^Gut Microbes and Health Institute Strategic Program, Quadram Institute Bioscience, Norwich, United Kingdom; ^2^Food Bioscience, Teagasc Food Research Centre Moorepark, Fermoy, Ireland; ^3^APC Microbiome Ireland, Teagasc and University College Cork, Cork, Ireland; ^4^Microhealth Group, Department of Microbiology and Biochemistry of Dairy Products, Instituto de Productos Lácteos de Asturias – Consejo Superior de Investigaciones Científicas, Villaviciosa, Spain

**Keywords:** *Staphylococcus epidermidis*, bile acids, biofilms, phylogeny, gastrointestinal tract, pangenome

## Abstract

*Staphylococcus epidermidis* is a commensal species that has been increasingly identified as a nosocomial agent. Despite the interest, little is known about the ability of *S. epidermidis* isolates to adapt to different ecological niches through comparisons at genotype or phenotype levels. One niche where *S. epidermidis* has been reported is the human gut. Here, we present three *S. epidermidis* strains isolated from feces and show that they are not phylogenetically distinct from *S. epidermidis* isolated from other human body sites. Both gut and skin strains harbored multiple genes associated with biofilm formation and showed similar levels of biofilm formation on abiotic surfaces. High-throughput physiological tests using the BIOLOG technology showed no major metabolic differences between isolates from stool, skin, or cheese, while an isolate from bovine mastitis showed more phenotypic variation. Gut and skin isolates showed the ability to metabolize glycine-conjugated bile acids and to grow in the presence of bile, but the gut isolates exhibited faster anaerobic growth compared to isolates of skin origin.

## Introduction

*Staphylococcus epidermidis* is a member of the coagulase-negative staphylococci (CoNS), considered a commensal and frequently found as a component of the human microbiome. *S. epidermidis* has been also identified as a nosocomial pathogen responsible for antibiotic resistant infections, isolated from catheters, bloodstream, prosthetic joints, ocular, and mastitis infections (Akinkunmi et al., 2014). This bacterium is highly adaptive and can associate with diverse hosts, including sheep, rodents, and plants (Chaudhry and Patil, 2016; Silverman et al., 2017). Despite the range of ecological niches, there is a pointed lack of comparative and evolutionary studies of ecologically diverse *S. epidermidis* isolates (Chaudhry and Patil, 2016). Within the human body, *S. epidermidis* has been found in the gastrointestinal (GI) tract (Akinkunmi et al., 2014). Furthermore, a recent study has identified a variety of gut pathogens, including *S. epidermidis*, in bloodstream infections, suggesting the GI tract as a source of those infections (Tamburini et al., 2018). This highlights the importance of identifying traits that could confer infection potential on *S. epidermidis* isolated from patients, for accurate tracking and treatment, as well as potentially revealing opportunities for disease prevention. However, public databases do not contain complete genomes of *S. epidermidis* isolates of gut/stool origin and little is known about distinct features that *S. epidermidis* of this origin may possess.

There are many factors which influence the composition and stability of microbes in the gut including pH, anaerobiosis, water activity, nutrients, and the presence and concentration of compounds such as bile acids (BA). It has been noted that bacteria adapted to live in the mammalian gut have developed strategies to overcome the antimicrobial activity of BAs, such as efflux systems, production of bile salt hydrolases (BSH) or remodeling of the cell wall (Urdaneta and Casadesús, 2017).

Some lineages of *S. epidermidis* can cause infections, a phenomenon which has been associated with an increased density of antimicrobial resistance and virulence-related genes in the genomes of those disease-causing lineages (Soeorg et al., 2017). Such *S. epidermidis* have been shown to cause pathological changes in the kidney and liver of rats and mice, among other organs (Akinkunmi et al., 2014). Other features associated with the ability of *S. epidermidis* to colonize include lipase activity, as this species is found mainly in lipid-rich regions (gut, skin, milk, etc.) and biofilm formation. Indeed, biofilm formation is considered one of the most important traits in *S. epidermidis* colonization, with a number of genes involved in the molecular mechanisms of the different stages of biofilm formation, such as adhesins involved in initial attachment, polysaccharide intercellular adhesin (PIA) matrix components involved in accumulation, teichoic acids in maturation and proteases and modulins in detachment (Arciola et al., 2012; Soumya et al., 2017). Taken together, there is evidence of traits that could reflect the ability of different strains of *S. epidermidis* to colonize different parts of the human body and suggest that pan-genome studies, such as that which proposed formate dehydrogenase as a potential clinical biomarker of pathogenesis in *S. epidermidis* (Conlan et al., 2012), have the potential to be of great value.

Here we study the pan-genome of *S. epidermidis*, including three new isolates of GI origin, with a view to identifying niche-specific traits. We also further investigate the existence of traits associated with lifestyle adaptation to the human gut, such as growth in anaerobic conditions and the presence of BA and potential pathogenic traits, like biofilm formation, in these strains.

## Materials and Methods

### Bacterial Strains and Culture Conditions

*S. epidermidis* F530B, YI07G and 9^c^ were isolated from a single fecal sample of a healthy individual from a study approved by the QIB Human Research Governance committee (IFR01/2015) registered at http://www.clinicaltrials.gov (NCT02653001). They were initially isolated and further grown in BHI media (Oxoid, United Kingdom) under aerobic and anaerobic conditions. We also used human skin isolates *S. epidermidis* DSM 20042 (DSMZ, Germany; tropical superficial axillary dermatitis isolate; grown in BHI under aerobic and anaerobic conditions) and *S. epidermidis* DSM 28764 (patient skin, Germany; BHI, aerobic and anaerobic); cheese isolate *S. epidermidis* DPC6293 (Teagasc culture collection, BHI, aerobic), and bovine mastitis isolate *S. epidermidis* DPC6010 (BHI, aerobic); positive control for biofilm formation *S. epidermidis* RP62A (ATCC 35984, isolated from an intravascular catheter-associated sepsis; BHI, aerobic and anaerobic), negative control for biofilm formation *S. epidermidis* ATCC 12228 [source unknown, non-infection related (Zhang et al., 2003), BHI, aerobic and anaerobic], positive control for BSH activity *Lactobacillus reuteri* NCIMB 30242 (MRS, aerobic and anaerobic) and negative control for survival ability in presence of bile *Escherichia coli* ATCC 25922 (LB, aerobic and anaerobic). All cultures were grown without shaking and at 37°C, unless otherwise stated.

### Isolation, Identification, and WGS

Fecal isolates were identified using 16S rRNA sequencing with PCR using primers F: 53′-GAG AGT TTG ATY CTG GCT CAG-33′ and R: 53′-AAG GAG GTG ATC CAR CCG CA-33′, product size of 1.5 kb (Baker et al., 2003) (Sigma) and GoTaq G2 polymerase (Promega). Whole genome sequence was provided by MicrobesNG (Birmingham, United Kingdom) using Illumina^®^ HiSeq and a 250 bp paired end protocol. Genome coverage was 30×. Reads were trimmed using Trimmomatic 0.30 with a sliding window quality cutoff of Q15 (Bolger et al., 2014) and the quality was assessed using software Samtools (Li et al., 2009), BedTools (Quinlan and Hall, 2010), and BWA mem (Li and Durbin, 2010). Reads for each sample were assembled with SPAdes V.3.7 (Bankevich et al., 2012), and the resulting contigs were scaffolded using Medusa (Bosi et al., 2015) with *S. epidermidis* ATCC 12228 as the reference genome. Genomes were annotated using Prokka 1.11 (Seemann, 2014). *S. epidermidis* DSM 20042 and *S. epidermidis* DSM 28764 were sequenced using Illumina MIseq V3 and their genomes were covered 40×. Reads were assembled and annotated using Prokka (v. 1.11).

### Genome Data Collection

All available *S. epidermidis* genomes were downloaded from the RefSeq genome database on 04/07/18 and metadata was downloaded from PATRIC database on 05/02/2019. 103 *S. epidermidis* metagenome-assembled genomes (MAGs) recovered from metagenomic data by Pasolli et al. (2019) were downloaded from http://segatalab.cibio.unitn.it/data/Pasolli_et_al.html. Two of these, OhJ_MET0231_bin_10 and ChngKR_WBU008_bin_2, were removed from the dataset as they showed low average nucleotide identity to the species representative genome *S. epidermidis* ATCC 12228 (79.4 and 79.75%, respectively). To have a consistent annotation, these genomes were annotated using Prokka (v. 1.14) and pan-genome-wide associations were performed using Roary (Page et al., 2015) and Scoary (Brynildsrud et al., 2016) with default parameters. The phylogenetic tree was constructed using PhyloPhlAn (v. 0.99) (Segata et al., 2013) and visualized using GraPhlAn (Asnicar et al., 2015). PlasmidFinder 2.0 (Carattoli et al., 2014) was used to identify potential plasmids. Blast 2.8.1 was used to search for stool-associated sequences in MAGs (Altschul et al., 1990).

### Phenotypic Characterization and Data Analysis

Phenotypic characterization was conducted using an OmniLog^®^ Phenotype Microarray^TM^ (Biolog, Inc., Hayward, CA, United States), following manufacturer’s instructions. PM1, PM2A, PM3B and PM11C (TECHNOPATH Distribution Ltd., Ireland) were incubated for 24 h. The data was analyzed by determining the area under the curve measured by OmniLog^®^ units.

### Bile Acids

#### Sample Preparation

*S. epidermidis* DSM 20042, DSM 28764, 9^c^ and F530B were grown in triplicate in 20 ml of BHI supplemented with 0.3% v/v porcine bile (Sigma, United Kingdom). One milliliter of each culture was collected at 24 and 48 h and kept at −20°C for further analysis. Solid phase extraction (SPE) clean-up was performed using Waters Oasis Prime HLB 1 30 mg SPE cartridges in a SPE vacuum system and washed with 1 ml of 5% methanol. Elution was performed with 500 μl 100% methanol using the same procedure and 25 μl of internal standards for each BA (Steraloids, United States) were added. The final volume was transferred to low volume autosampler tubes for liquid chromatography-mass spectrometry (LC-MS) analysis.

#### Liquid Chromatography-Mass Spectrometry

Clean extracts were analyzed using LC-MS operated in multiple reaction monitoring (MRM) mode. Each sample (5 μl) was analyzed using an Agilent 1260 binary HPLC coupled to an AB Sciex 4000 QTrap triple quadrupole mass spectrometer. HPLC was achieved using a binary gradient of solvent A (water + 5 mM ammonium acetate + 0.012% formic acid) and solvent B (methanol + 5 mM ammonium acetate + 0.012% formic acid) at a constant flow rate of 600 μl/min. Separation was made using a Supelco Ascentis Express C18 150 × 4.6, 2.7 μm column maintained at 40°C. Injection was performed at 50% B and held for 2 min, ramped to 95% B at 20 min and held until 24 min. The column equilibrated to initial conditions for 5 min. The mass spectrometer was operated in electrospray negative mode with capillary voltage of −4500 V at 550°C. Instrument specific gas flow rates were 25 ml/min curtain gas, GS1: 40 ml/min and GS2: 50 ml/min. Quantification was applied using Analyst 1.6.2 software to integrate detected peak areas relative to the deuterated internal standards.

#### Growth Curves

Growth behavior of *S. epidermidis* DSM 20042, DSM 28764, 9^c^, F530B, RP62A, ATCC 12228, *L. reuteri* NCIMB 30242 and *E. coli* ATCC 25922 was monitored aerobically and anaerobically. Twenty milliliter of BHI was inoculated with 1% of each studied strain with or without 0.3% porcine bile and triplicate 300 μl aliquots were transferred to a honeycomb^®^ sterile plate (Thermo Fisher Scientific, United Kingdom) for Bioscreen C (Labsystems Diagnostics Oy, Finland) for measuring aerobic growth [optical density (OD) at 600 nm] and to a 96-well plate for Infinite F50 (Tecan Group, Switzerland) for anaerobic conditions, using Bioscreener and Gen5 Data Analysis as software, respectively. Growth rate (μ) was calculated using the formula μ = 2.303 (log OD_2_ − log OD_1_)/*t*_2_ − *t*_1_ (Ng et al., 2015) where OD_2_ is the value that doubles OD_1_, and *t*_2_ and *t*_1_ the time at those two measurements.

### Biofilm Formation on Abiotic Surfaces

To measure biofilm formation, bacterial cultures were standardized for 2 days. A colony of each bacteria was cultured in 10 ml BHI. After 24 h, it was subcultured in 10 ml fresh medium at 1% and another subculture was performed after another 24 h. Bacterial counts were performed and 200 μl or 1 ml of bacterial culture was added to each well of a 96-well polystyrene (PE) plate or a 12-well plate (Falcon, Becton Dickinson) with a sterilized glass cover, respectively. After 24 h incubation at 37°C, supernatants were removed from the wells which were then washed with equivalent volumes of PBS. One volume of 0.1% crystal violet (Sigma, United Kingdom) was added and incubated at room temperature for 15 min. Crystal violet was removed from the wells and an equivalent volume of 33% acetic acid was added. The mixture was transferred immediately to a 96-well plate and OD read at λ = 595 nm.

### Statistical Analysis

Significant differences between groups were established using a paired *t*-test, assuming normal distribution, equal variances. Both sides of the distribution were considered. Comparison of genes in MAGS was performed using a Welch Two Sample *t*-test. Differences were considered to be significant when the *p* value was <0.05. Calculations were performed using Excel 365. The Benjamini–Hochberg procedure was used to control type 1 errors when performing pangenome-wide association using Scoary and reduce the number of significant associations which may have occurred by chance.

## Results

### *S. epidermidis* Pan-Genome

**FIGURE 1:**
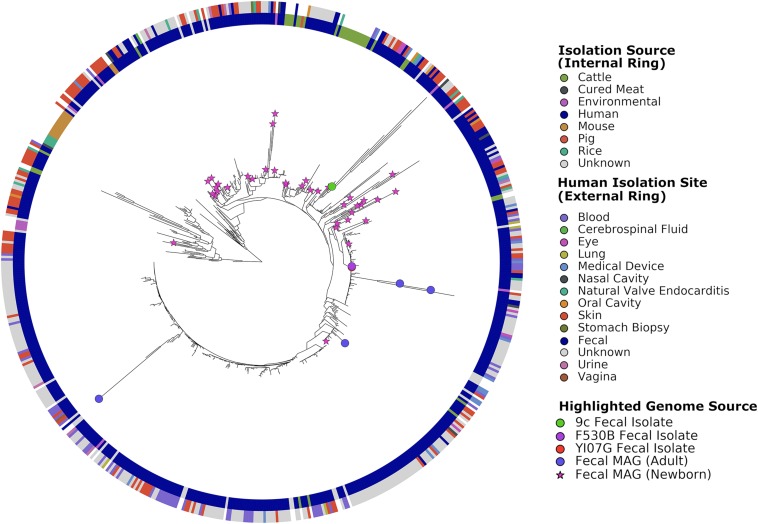
Phylogenetic tree of all genomes used in this study constructed using PhyloPhlAn. Leaves representing fecal-associated genomes are highlighted on the tree, reference genomes and MAGs of non-fecal origin are not highlighted for readability. The inner ring shows the source of the isolated genomes or samples from which MAGs were recovered. The outer ring shows the body site from which the genome or MAG was isolated or recovered.

The *S. epidermidis* pan-genome was determined with Roary, which clusters the protein-encoding genes into core (hard and soft) and accessory (shell and cloud) genes. The pan-genome contained 18,436 total genes across 549 genomes: 713 core genes present in ≥99% of genomes, 443 soft core genes present in 95–99% of genomes, 1,823 shell genes present in 15–95% of genomes, and 15,457 cloud genes present in less than 15% of genomes. To visualize results, phylogenetic trees were constructed, included in these were the 101 MAGs recovered by Pasolli et al. (2019) (Figure 1). Stool-associated MAGs did not show clustering. The three stool isolates did not cluster together, with 9^c^ (accession number WLUZ00000000) being the one that was most different. However, two – *S. epidermidis* F530B (accession number WITG00000000) and YI07G (accession number WLVA00000000)-overlapped and were located relatively closely to two MAGs recovered from adult stool samples. The remaining stool isolate located on a region of the tree which suggests it is more phylogenetically related to skin isolates and MAGs recovered from newborn stool samples. Cattle isolates did not form a single cluster but did show some slight localization, while rice and murine isolates exhibited strong phylogenetic clustering. The genomes from different human body sites isolates did not cluster, suggesting no specialization.

Comparison of the three stool isolate genomes gave pairwise average nucleotide identities of 99.44% (*S. epidermidis* 9^c^ : F530B), 99.41% (*S. epidermidis* 9^c^ : YI07G) and 99.96% (*S. epidermidis* F530B : YI07G). Scoary identified 44 genes whose presence was significantly associated with being isolated from human stool (Table 1). Twenty-eight of these 44 ORFs were located on a 30 kb contiguous sequence which was identified as a probable plasmid by PlasmidFinder2.0, suggesting the likely acquisition of a horizontally transmitted genetic element conferring the ability to survive in the human GI tract. Searching for these genes in the MAGs using Blastn did not show a significantly greater number in fecal MAGs compared to those from other human body sites (Welch Two Sample *t*-test, *p* = 0.8141), nor was it possible to identify the potential plasmid sequence in any MAGs using Blastn. Although not significantly associated, all three stool isolates contain a type V arginine catabolic module element (ACME).

**TABLE 1:** Genes significantly correlated with gut-associated *S. epidermidis* isolates.

**Contig accession**	**Gene accession number**	**Gene name/group**	*p*-value*	**Blastp match**	**Blastp Identity (%)**	**Query coverage (%)**
WITG01000003	GQY01_RS05830	group_464	0.01788	ISSag7, transposase OrfA [*S.epidermidis* VCU045]	100	100
	GQY01_RS05840	*sdrG*	0.04113	MSCRAMM family cell wall-anchored protein SesJ [*S. epidermidis*]	93.19	100
	GQY01_RS05845	group_4391	0.01151	lipopolysaccharide biosynthesis protein [*S. epidermidis*]	100	100
	GQY01_RS06000	group_12034	0.01506	hypothetical protein [*Staphylococcus*]	100	100
	GQY01_RS06005	*hsdR_1*	0.01788	type I restriction endonuclease subunit R [*Staphylococcus*]	99.80	100
	GQY01_RS06010	group_9501	0.01151	restriction endonuclease subunit S [*Staphylococcus*]	100	100
	GQY01_RS06015	*hsdM_1*	0.01643	SAM-dependent DNA methyltransferase [Staphylococcus]	100	100
	GQY01_RS06020	group_10501	0.01329	hypothetical protein SEVCU037_0543 [*S. epidermidis* VCU037]	100	100
	GQY01_RS06025	group_12033	0.01506	DUF1643 domain-containing protein [*S. epidermidis*]	100	100
	GQY01_RS06035	group_317	0.01643	tandem lipoprotein [*S. epidermidis* VCU045]	100	100
	GQY01_RS06075	group_6301	0.04732	type III-A CRISPR-associated RAMP protein Csm5 [*Staphylococcus*]	100	100
	GQY01_RS06085	*cas6*	0.04732	CRISPR-associated endoribonuclease Cas6 [*Staphylococcus*]	100	100
WITG01000014	GQY01_RS11390	group_10720	0.01329	**metal-dependent transcriptional regulator [*S. epidermidis*]**	100	100
	GQY01_RS11395	*fecE_2*	0.01329	**metal ABC transporter ATP-binding protein [*Staphylococcus*]**	99.59	100
	GQY01_RS11400	*mntB_2*	0.01329	**metal ABC transporter permease [*Staphylococcus*]**	100	100
	GQY01_RS11405	*mntA_2*	0.01329	**zinc ABC transporter substrate-binding protein [*Staphylococcus*]**	100	100
	GQY01_RS11410	group_12447	0.01329	**recombinase family protein [*Staphylococcus*]**	100	100
	GQY01_RS11415	group_13561	0.00155	**hypothetical protein [*Staphylococcus*]**	99.85	100
	GQY01_RS11420	group_13560	0.00155	**hypothetical protein [*Staphylococcus*]**	100	100
	GQY01_RS11430	group_13559	0.00110	**NERD domain-containing protein [*Staphylococcus*]**	100	100
	GQY01_RS11440	group_13558	0.00110	**hypothetical protein HMPREF2913_01140 [*Staphylococcus* sp. HMSC065A08]**	94.74	100
	GQY01_RS11445	group_13154	0.01329	**poly(glycerol-phosphate) alpha-glucosyltransferase [*Staphylococcus*]**	99.44	100
	GQY01_RS11450	*sdrI_1*	0.04732	**YSIRK signal domain/LPXTG anchor domain surface protein [*S. epidermidis*]**	97.94	94
	GQY01_RS03690	*sdrI_2*	9.49E-05	**YSIRK-type signal peptide-containing protein [*S. epidermidis*]**	87.93	100
	GQY01_RS11455	group_9826	0.01000	**helix-turn-helix domain-containing protein [*S. aureus*]**	85.71	100
	c(15152..15343)	group_16636	9.49E-05	**hypothetical protein HMPREF2566_04185 [*Staphylococcus* sp. HMSC070A07]**	100	100
	GQY01_RS11460	group_16637	9.49E-05	**no significant similarity found**		
	GQY01_RS11465	group_16638	9.49E-05	**hypothetical protein [*Staphylococcus*]**	100	100
	GQY01_RS11470	group_16639	9.49E-05	**peptidase domain-containing ABC transporter [*Staphylococcus*]**	100	100
	GQY01_RS11475	group_16640	9.49E-05	**DsbA family protein [*Staphylococcus*]**	100	100
	c(19667..19813)	group_10569	0.01643	**hypothetical protein [*S. haemolyticus*]**	97.92	100
	GQY01_RS11480	group_503	0.02693	**replication initiator protein A [*Staphylococcus*]**	100	100
	GQY01_RS11485	*racA*	9.49E-05	**DUF536 domain-containing protein [*Staphylococcus*]**	100	100
	GQY01_RS11495	group_588	0.00155	**RepB family plasmid replication initiator protein [*Staphylococcus* sp. HMSC070A07]**	100	100
	GQY01_RS11500	*fas6*	0.02047	**TIGR00730 family Rossman fold protein [*Staphylococcus*]**	100	100
	GQY01_RS11505	group_11017	0.00110	**hypothetical protein [*Staphylococcus*]**	100	100
	GQY01_RS11515	group_11121	0.00033	**hypothetical protein [*Staphylococcus* sp. HMSC068G11]**	100	57
	GQY01_RS11520	group_7970	0.00232	**hypothetical protein [*Staphylococcus* sp. HMSC070A07]**	99.33	100
	GQY01_RS11530	group_2880	0.00812	**DUF3139 domain-containing protein [Bacteria]**	100	100
	GQY01_RS11535	group_4374	0.04113	**hypothetical protein [*Staphylococcus*]**	100	100
WITG01000019	GQY01_RS11920	group_481	0.01788	recombinase [*Staphylococcus*]	99.75	100
	c(4680..5045)	group_9397	0.00110	hypothetical protein [Bacilli]	100	100
	GQY01_RS11940	group_8755	0.00812	hypothetical protein B467_01449 [*S. epidermidis* M0881]	99.59	100
	GQY01_RS11945	group_12141	0.04732	hypothetical protein [Bacilli]	100	100

* Benjamini and Hochberg adjusted. Genes co-located on 30 kb contiguous sequence are highlighted. Locus ID corresponds to the genome of 5299-F530B. Contig coordinates of the open reading frame are provided where a locus ID was unavailable. Cluster of gene groups were defined with the pangenome analysis (Roary) using 549 *S. epidermidis* genomes. Protein sequences were searched against the NCBI’s non-redundant database to improve the annotation.

### Biolog Analysis of Nutrient Sources and Antibiotic Resistance

**FIGURE 2:**
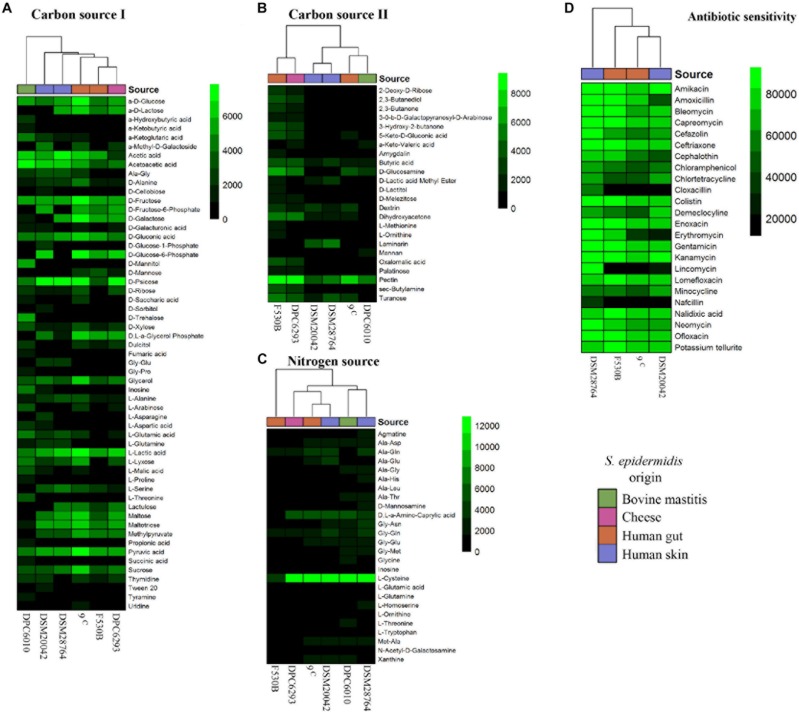
Biolog results for growth of *S. epidermidis* stool (9^c^ and F530B), skin (DSM 20042 and DSM 28764), cheese (DPC6293), and mastitis (DPC6010) isolates. **(A)** Carbon sources (I) PM1; **(B)** Carbon sources (II) PM2A; **(C)** Nitrogen sources PM3B; and **(D)** Antibiotic PM11C. Colors in scales represent arbitrary units of growth based on Omnilog growth units.

Two of the gut isolates which were well separated in the phylogenetic analysis, *S. epidermidis* F530B and 9c, were compared to two further human isolates from a different location (skin, *S. epidermidis* DSM 20042 and DSM 28764) and environmental isolates from cheese (*S. epidermidis* DPC6293) and bovine mastitis (*S. epidermidis* DPC6010), to compare the effect of isolation source on physiology. Phenotype experiments conducted using BIOLOG technology showed differences in the utilization of carbon and nitrogen substrates and antibiotic sensitivity between strains (Figure 2). The mastitis isolate *S. epidermidis* DPC6010 showed an ability to grow in a broader range of carbon sources than the other isolates. There was some variation in utilization from the other strains, but no clear delineation according to origin, and the pairs of gut and skin isolates often showed a different growth pattern.

Antibiotic sensitivity only showed one differential pattern between skin and stool isolates. The growth of gut isolates was inhibited by lincomycin, while it did not fully inhibit the growth of skin isolates. One skin isolate, *S. epidermidis* DSM 28764, showed growth in the presence of all antibiotics.

### Effect of Bile Acids on *S. epidermidis* Skin and Gut Isolates

#### Patterns of Growth *in vitro*

**FIGURE 3:**
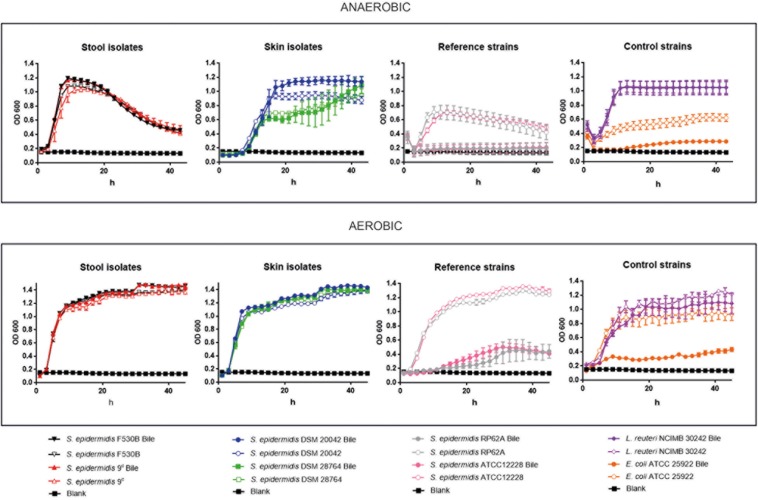
Growth curves in aerobic and anaerobic conditions, with and without bile, of stool isolates (*S. epidermidis* 9^c^ and F530B), skin isolates (*S. epidermidis* DSM 20042 and DSM 28764), sepsis-related *S. epidermidis* RP62A and non-infection related *S. epidermidis* ATCC 12228, *E. coli* ATCC 25922 for negative control and *L. reuteri* NCIMB 30242 with bile salt hydrolase activity for positive control. Results are the mean of three replicates ± standard deviation.

Growth curves of stool isolates *S. epidermidis* 9^c^ and F530B, and skin strains (*S. epidermidis* DSM 20042 and DSM 28764) was compared with two further *S. epidermidis* strains (RP62A, sepsis-related strain and ATCC 12228, a non-infection related strain), *E. coli* ATCC 25922 whose growth is attenuated in presence of bile salts (Sung et al., 1993) and *L. reuteri* NCIMB 30242, that possesses BSH activity. Growth assays were performed in the presence and absence of porcine bile and under aerobic and anaerobic conditions, to examine their ability to survive bile and grow with and without oxygen. When grown under aerobic conditions, stool isolates *S. epidermidis* 9^c^ and F530B and skin isolates *S. epidermidis* DSM 20042 and DSM 28764 exhibited similar exponential growth rates (Figure 3). They all showed the ability to grow in the presence of bile and their growth rate was not affected by it. *L. reuteri* NCIMB 30242 growth was also not negatively affected by bile. The two further *S. epidermidis* isolates, RP62A and ATCC 12228, had similar growth patterns to the other four *S. epidermidis* isolates in the absence of bile, but their growth was notably reduced in the presence of bile, in a similar way to *E. coli* ATCC 25922.

Under anaerobic conditions, differences were observed between gut and skin isolates (Figure 3). Stool strains *S. epidermidis* 9^c^ and F530B showed similar growth rates of 0.33 ± 0.01 and 0.32 ± 0.01 in the presence of bile and 0.31 ± 0.0 and 0.36 ± 0.0 without bile, respectively, with slightly higher maximum ODs with bile, and *L. reuteri* NCIMB 30242 also grew well in anaerobic conditions both with and without bile. Skin isolates *S. epidermidis* DSM 20042 and DSM 28764 showed a lag time of up to 4 h before growth, both strains then had similar growth rates with or without bile, but the exponential growth rates were lower than those of the gut isolates (0.15 ± 0.02 and 0.15 ± 0.06 with bile and 0.18 ± 0.0 and 0.15 ± 0.0 without bile, respectively). The OD of the gut isolates dropped over time once the maximum growth had been achieved, which might represent cell lysis or aggregation. Skin isolate *S. epidermidis* DSM 20042 maintained its OD over 48 h, with bile giving a final higher OD; *S. epidermidis* DSM 28764 grew more slowly and its OD continued to increase over 48 h, again with bile giving a slightly higher final OD. Although these two skin isolates need more time to reach their maximum growth in anaerobic conditions, they eventually achieve the same maximum ODs as the gut isolates when supplemented with bile. In contrast, isolates *S. epidermidis* RP62A and ATCC 12228 were more clearly affected by anaerobic conditions, showing reduced growth with no bile and attenuated or no growth in bile without oxygen.

#### Bile Acids Quantification

**FIGURE 4:**
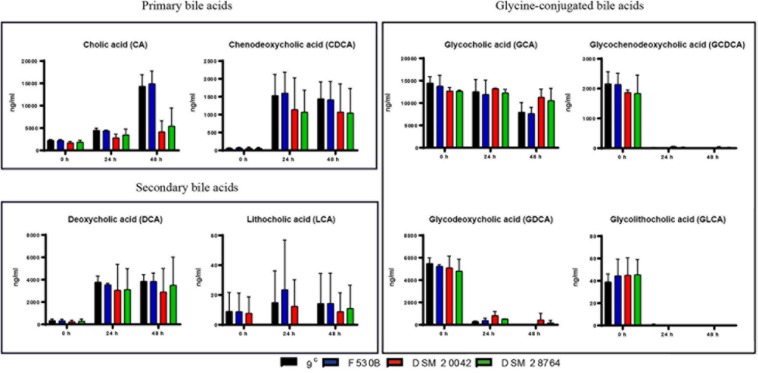
Primary, secondary and glycine-conjugated bile acids in the media after fermentations of *S. epidermidis* gut isolates 9^c^ and F530B and skin isolates DSM 20042 and DSM 28764. Results (ng/ml) are the mean of duplicate measurements +/− standard deviation. Media control values remain unaltered through time and are not represented.

The degradation of cholesterol gives primary bile acids, cholic acid (CA) and chenodeoxycholic acid (CDCA) that are later transformed by intestinal bacteria into secondary BAs by removing a hydroxyl group from the C7 of the primary BA molecule, giving deoxycholic acid (DCA) from CA and lithocholic acid (LCA) from CDCA. Bile also contains BAs conjugated with glycine and taurine. Bile acids are commonly very stable and incubations in the absence of bacteria had no effect on composition. However, when gut and skin isolates of *S. epidermidis* were grown with porcine bile, we detected a progressive increase of primary bile acids CA and CDCA with time, while concentrations of secondary bile acids, DCA and to some extent LCA, also increased. The concentrations of DCA in the culture of skin isolates were very variable, while the much lower levels of LCA varied widely in all isolates and it was not detectable in the culture of *S. epidermidis* DSM 28764 until 48 h (Figure 4). There is a large decrease at 24 h of glycine conjugated primary and secondary bile acids glycochenodeoxycholic acid (GCDCA), glycodeoxycholic acid (GDCA) and glycolithocholic acid (GLCA), which somewhat mirrored the rise in deconjugated acids for CDCA and DCA. The glycocholic acid (GCA) levels did not show as sharp a decrease as the others. The levels of the taurine conjugated BAs remained constant over time in *S. epidermidis* cultures of both skin and gut origins (data not shown).

### Biofilm-Forming Abilities

#### Genomic Comparison

The comparison of the genes involved in biofilm development, previously described as virulence factors, in *S. epidermidis* 9^c^ and F530B (gut), DSM 20042 and DSM 28764 (skin) showed no major differences. Only two genes of potential relevance, *sdrC_2*, encoding Ser-Asp rich fibrinogen/bone sialoprotein-binding protein SdrC and *clfB*, encoding clumping factor B, were present in the genomes of gut isolates and absent in the genomes of skin isolates (Table 2). Both genes are related to the accumulation stage of biofilms, involved in cell-cell aggregation and matrix production. The SdrC protein has been highlighted as being a determinant of staphylococcal biofilm formation (Barbu et al., 2014). *Ica* genes, characteristic *Staphylococcus* genes involved in biofilm formation, were present in the genome of all four isolates, as were other genes involved in the biofilm maturation stage and related to thickness, such as teichoic acid biosynthesis proteins. The *agr* operon, involved in quorum sensing control of biofilm formation, was incomplete in both gut and skin isolates, which all lacked *agrC*.

**TABLE 2:** Comparison of presence of genes involved in biofilm formation in *S. epidermidis* stool isolates 9^c^ and F530B and skin strains DSM 20042 and DSM 28764.

**Gene Accession number**	**Biofilm function Gene annotation**		**9^c^**	**F530B**	**DSM 20042**	**DSM 28764**
**1. Initial attachment. Adhesion to material surface**						
GQY01_RS08455	*atl*	bifunctional N-acetylmuramoyl-L-alanine amidase/endo-beta-N-acetylglucosaminidase, Atl	√	√	√	√
**2. Accumulation. Cell-cell aggregation, matrix production**						
GQY01_RS12155	*icaD*	intercellular adhesion protein IcaD	√	√	√	√
SERP2296	*icaC*	intercellular adhesion protein icaC	√		√	√
SERP2295	*icaB*	intercellular adhesion deacetylase IcaB	√		√	√
SERP2293	*icaA*	intercellular adhesion protein icaA	√		√	√
GQY01_RS05560	*sdrD*	Ser-Asp rich fibrinogen/bone sialoprotein-binding protein SdrD	√	√	√	√
GQY01_RS05840	*sdrC*	Ser-Asp rich fibrinogen/bone sialoprotein-binding protein SdrC	√	√	√	
GQY01_RS09660	*sdrH*	Ser-Asp rich fibrinogen/bone sialoprotein-binding protein SdrH	√	√	√	
GQY01_RS10940	*sdrD_1*	Ser-Asp rich fibrinogen/bone sialoprotein-binding protein SdrE		√	√	√
GQY01_RS09245	*sdrE_1*	Ser-Asp rich fibrinogen/bone sialoprotein-binding protein SdrE_1		√		√
GQY01_RS00755	group_61	cell-wall-anchored protein SasC	√	√	√	
GQY01_RS11450	*sdrC_2*	Ser-Asp rich fibrinogen/bone sialoprotein-binding protein SdrC	√	√		
AJ224764	*clfB*	clumping factor B	√	√		
**3. Maturation. Thickness increase, dependence of structural features**						
GQY01_RS09690	*agrA*	autoinducer sensor protein response regulator protein	√	√	√	√
GQY01_RS09675	*agrB*	accessory gene regulator B	√	√	√	
GQY01_RS09680	*agrD*	staphylococcal accessory gene regulator protein D	√	√	√	
GQY01_RS07235	*wecG*	teichoic acid biosynthesis protein	√	√	√	√
GQY01_RS07225	*tagG*	teichoic acid ABC superfamily ATP binding cassette transporter, membrane protein	√	√	√	√
GQY01_RS04020	group_2824	D-Ala-teichoic acid biosynthesis protein	√	√	√	√
GQY01_RS07230	*tagH*	Teichoic acid export ATP-binding protein, TagH	√	√	√	√
GQY01_RS07220	*tagB*	teichoic acid biosynthesis protein B	√	√	√	√
GQY01_RS07215	*tagX*	teichoic acid biosynthesis protein X	√	√	√	√
GQY01_RS09275	group_3224	teichoic acid ABC superfamily ATP binding cassette transporter, membrane protein	√	√	√	√
GQY01_RS04000	*dltD*	D-alanine lipoteichoic acid and wall teichoic acid esterification secreted protein	√	√	√	√
**4. Regulation**						
GQY01_RS10450	*rsbU*	putative sigma factor sigB regulation protein	√	√	v	√
GQY01_RS07335	*sarA*	staphylococcal accessory regulator family protein	√	√	√	√
GQY01_RS10440	*rsbW*	anti-sigma-B factor, serine-protein kinase	√	√	√	√
GQY01_RS10435	*sigB*	RNA polymerase sigma factor SigB	√	√	√	√
GQY01_RS10445	*rsbV*	anti-sigma-B factor, antagonist	√	√	√	√
GQY01_RS02455	*msa*	modulator of SarA, Msa	√	√		√
**5. Other genes involved in biofilm formation**						
GQY01_RS00765	group_2411	putative polysaccharide biosynthesis protein	√	√	√	√
GQY01_RS02140	group_2526	lipopolysaccharide biosynthesis-related pr-like protein	√	√	√	√
GQY01_RS03710	*kdtB*	lipopolysaccharide core biosynthesis protein KdtB	√	√	√	√
GQY01_RS03810	*paaD*	putative metal-sulfur cluster biosynthetic enzyme, PaaD	√	√	√	√
GQY01_RS10015	*fmhB*	peptidoglycan pentaglycine interpeptide biosynthetic protein FmhB	√	√	√	√
GQY01_RS03745	group_3157	SCP/PR1-like extracellular protein	√	√	√	√
GQY01_RS04625	*fmhA*	FemAB family peptidoglycan biosynthesis protein	√	√	√	√
GQY01_RS10015	*fmhB_2*	peptidoglycan pentaglycine interpeptide biosynthetic protein FmhB	√	√		

*Cluster of gene groups were defined with the pangenome analysis (Roary) using the four *S. epidermidis* genomes.*

#### Biofilm Formation on Abiotic Surfaces

*S. epidermidis* strains F530B, 9^c^, DSM 20042 and DSM 28764 were able to form biofilms on abiotic surfaces, PE and glass, at 1:100 dilution, but a lower number of cells (1:1000 dilution) generally failed to form a measurable biofilm (Table 3). There were no significant differences between the four isolates. Biofilm formation was slightly lower than that seen with a known biofilm forming strain, *S. epidermidis* RP62A while a strain previously described as not forming biofilm, *S. epidermidis* ATCC 12228 (Zhang et al., 2003), gave notably lower levels of crystal violet staining on both glass and PE (Table 3).

**TABLE 3:** Biofilm formation of *S. epidermidis* measured by crystal violet staining on glass and PE.

	**Glass**		**PE**	
**Dilution**	**1:100**	**1:1000**	**1:100**	**1:1000**
*S. epidermidis* F530B	0.531 ± 0.027	0.026 ± 0.016	0.712 ± 0.027	0.032 ± 0.007
*S. epidermidis* 9^c^	0.483 ± 0.104	0.011 ± 0.009	0.698 ± 0.113	0.045 ± 0.011
*S. epidermidis* DSM 20042	0.512 ± 0.085	0.012 ± 0.006	0.675 ± 0.051	0.012 ± 0.003
*S. epidermidis* DSM 28764	0.611 ± 0.077	0.082 ± 0.032	0.669 ± 0.095	0.033 ± 0.008
*S. epidermidis* RP62A	0.683 ± 0.081	0.039 ± 0.013	0.796 ± 0.067	0.039 ± 0.015
*S. epidermidis* ATCC 12228	0.156 ± 0.081	0.092 ± 0.01	0.214 ± 0.087	0.088 ± 0.019
Media controls	0.063 ± 0.001	0.057 ± 0.002	0.054 ± 0.002	0.059 ± 0.002

*Figures are the means of three measurements ± standard deviations.*

## Discussion

In this study we have presented three *S. epidermidis* strains isolated from the human gut, a source which, to our knowledge, is not represented in online databases. We have placed these strains in an updated *S. epidermidis* phylogenetic tree and further looked for genomic traits that differentiate these from *S. epidermidis* isolated from other sources, such as human body sites and other environmental niches. We have also investigated phenotypic differences between these strains and other *S. epidermidis* isolated from human skin, a case of bovine mastitis and cheese, specifically in terms of utilization of carbon and nitrogen sources and antibiotic sensitivity, and we have associated these differences with genomic information in strains of human origin. Finally, we have compared the behavior of these strains in terms of traits that could influence their lifestyles, such as growth in the presence of bile and virulence traits such as biofilm formation.

While there was limited phylogenetic clustering of gut-associated *S. epidermidis* isolates and MAGs, other strains with a common origin exhibited clustering, such as those isolated from rice and mice. However, the rice and mice isolates were obtained in the same study (Chaudhry and Patil, 2016) and likely represent a single geographic area. *S. epidermidis* isolates from different sites in and on the human body appeared to be more variable than their non-human counterparts, a phenomenon that has been reported previously for *S. epidermidis* (Becker et al., 2014). Interestingly, phylogenetic similarity between MAGs recovered from newborn fecal samples and skin isolates suggests that the presence of *S. epidermidis* in infant feces may be transient and transmitted by skin contact during breast feeding. This may also explain why one isolate, 9^c^, appeared to be phylogenetically more similar to skin isolates than the other fecal isolates. One recent study by Zhou et al. (2020), reported substantial transmission of *S. epidermidis* strains between facial sites and other skin sites, particularly the hands and antecubital fossa, across all participants. Although no fecal samples were screened in that study, it is possible that touching of the face and mouth area may offer a route of transmission from the skin to the gut. One pan-genome analysis conducted in 2012 examining skin commensal and hospital infection-associated *S. epidermidis* showed high levels of intra-site diversity and two clear phylogenetic groups which differentiated the commensal and the nosocomial isolates (Conlan et al., 2012). A second whole-genome comparison study of *S. epidermidis* reported that rice endophytic strains are more related to rodent isolates than the majority of human isolates, an observation which is also reported using our approach (Chaudhry and Patil, 2016). These two studies were conducted on 99 and 93 genomes, respectively, while our pan-genome approach used 549 genomes.

While the three gut isolates described here do not form a distinct phylogenetic cluster, our pan-genome-wide association approach was able to identify 44 genes, in four clusters, significantly associated with gut isolates, including a predicted 30 kb plasmid. Twenty-eight of the 30 ORFs on that plasmid were significantly associated with stool isolates and encode some genes involved in metal metabolism, cell surface components, resistance mechanisms, transporters, and transcriptional regulators. In addition, we also report that all three gut isolates contain a type V ACME gene cluster characterized by the presence of the arginine deaminase pathway-encoding *arc* operon, the oligopeptide permease ABC transporter-encoding *opp*3 operon, and the potassium transport system-encoding *kdp* operon (O’Connor et al., 2018a). These ACME gene clusters play a role in colonization of the host and evasion of the immune system (Diep et al., 2008; Planet et al., 2013), have been previously described in *S. epidermidis* isolated from the human skin and oral cavity (Planet et al., 2013; O’Connor et al., 2018a), and are significantly enriched in infected subgingival oral implants compared to non-infected counterparts (O’Connor et al., 2018b).

These results led us to study possible phenotypic differences between gut and skin *S. epidermidis* isolates. Observations made from the simplified phylogenetic tree led us to select strains isolated from bovine mastitis and cheese for comparison. We showed that there were some differences in the use of carbon sources between *S. epidermidis* of different origin, with the isolate from bovine mastitis showing the most variation, but there was no clear separation related to isolate origin for cheese, skin and gut strains. Combining the phenotypic and pan-genomic data for these strains, where available, indicated that utilization of laminarin was associated with 22 genes including merABR, involved in mercury resistance, and the formate reductase-encoding *fdh*D gene which has been previously observed to be only present in commensal *S. epidermidis* and touted as a potential biomarker to discriminate between harmless strains and those associated with hospital-acquired infections (Conlan et al., 2012). Despite a small sample size, these carbon sources can be pointed to for further research as having potential for identifying the origin of *S. epidermidis* isolates and designing better treatments for nosocomial infections. The nitrogen usage for the different *S. epidermidis* isolates did not show major differences that could be associated to the isolate origin, as with the carbon sources. However, many studies examined the nitrogen usage of *S. epidermis* (Emmett and Kloos, 1975; Austin et al., 2012). *Staphylococcus* spp. have been proved extensively as being able to be trained to use different nitrogen sources by reorganizing their metabolism to the extent of being able to use ammonia as a nitrogen source instead of amino acids (Gladstone, 1937; Knight, 1937). Despite the highly conserved core genome of *Staphylococcus* spp metabolism (Bosi et al., 2016), they can show strain-specific metabolic adaptations (Heinemann et al., 2005; Lee et al., 2009; Bosi et al., 2016).

No BSH genes were identified in the genomes of stool and skin isolates. However, we could observe that there was a change in the concentrations of BAs in the culture media of both stool and skin isolates. With both gut and skin isolates, the glycine bond to the steroid nucleus of the bile was removed (deconjugation) in GCDCA, GDCA, and GLCA, a process that increases bile tolerance and survival in the gut and is the pre-requisite for biotransformations in human colon, where DCA and LCA are the predominant BS in human feces (Urdaneta and Casadesús, 2017). When glycine and taurine are separated from the BS, they can be used as sources of C and N (Ridlon et al., 2016). The deconjugation is catalyzed by the BSH and, despite the fact that no BSH were identified in the genes, it is likely that some *S. epidermidis* genomes encode enzymes able to perform deconjugation. These results, in combination with the growth curves observed in anaerobic conditions, suggest that some skin isolates are able to behave similarly to gut isolates but need some time to adapt to the absence of oxygen. Conversely, *S. epidermidis* ATCC 12228 and RP62A – a non-infection related and a sepsis-related strain, respectively – showed less ability to grow in anaerobic conditions and clearly attenuated growth in bile. Once the skin isolates *S. epidermidis* DSM 20042 and DSM 28764 start growing, bile does not have a clear impact on exponential growth. Interestingly, the growth displayed by the skin isolates in the presence of BA suggest that they may use these BA as a source of nutrients in anaerobic conditions, as there seems to be a positive effect on final OD. The decrease of glycine-conjugated BAs might suggest that the glycine could have been used. In contrast, taurine-conjugated BAs were not metabolized by any isolate, which is consistent with the fact that only one intestinal species, *Bilophila wadsworthia*, is able to use taurine (Laue et al., 1997; Carbonero et al., 2012). Enzymes for biotransformations of CA into DCA and CDCA into LCA are not as widespread in nature as enzymes for deconjugation, and only a few Clostridia bacteria are able to remove the hydroxyl from the position 7 (Mallonee and Hylemon, 1996; Urdaneta and Casadesús, 2017). It has been reported, however, that some *Clostridium* and *Bacteroides* spp are able to use different metabolic routes that enhance bile salt degradation (Huijghebaert and Eyssen, 1982; Van Eldere et al., 1996; Ridlon et al., 2016). A significant rise in the concentration of DCA could be observed after incubation with *S. epidermidis*, which suggests BA transformation of some type. Further functional analyses, including transcriptomics, proteomics and metabolomics might help reveal a route to identify the enzymes and/or metabolic routes involved in this relationship process.

Other traits considered to be associated with virulence, like biofilm formation, showed similar patterns in stool and skin isolates. Small differences were observed at the genomic level, in that only two genes of known relevance – *sdrC_2* and *clfB* – were present in the genome of stool isolates and were absent from the skin isolates. *sdrC* has a second version present in one of the skin isolates which may provide redundancy. *Agr* genes are related to quorum sensing systems and their absence produces stronger *S. aureus* biofilms (Vuong et al., 2003). Here, some *agr* elements are missing from skin and gut isolates, but no differences were observed in biofilm formation in the conditions tested.

These results suggest the absence of major specific genomic traits among *S. epidermidis* for adaptation to the different sites within the human body. Therefore, *S. epidermidis* adaptation to human body sites might mainly rely on gene expression, and techniques such as transcriptomics, proteomics and metabolomics would be the next step to understand how the metabolism reorganizes to adapt to these sites, with care to avoid laboratory domestication. However, more cultures from diverse sampling sources would be required to strengthen these observations.

## Data Availability Statement

Sequencing data can be found on https://www.ncbi.nlm.nih.gov/genbank under the accession numbers WLUZ00000000, WITG00000000, and WLVA00000000.

## Ethics Statement

The studies involving human participants were reviewed and approved by the QIB Human Research Governance committee (IFR01/2015) registered at http://www.clinicaltrials.gov (NCT02653001). The patients/participants provided their written informed consent to participate in this study.

## Author Contributions

EG-G, CW, TD-C, MM, PC, and AN conceived the study. EG-G and DT performed the experiments. EG-G, CW, LS, TD-C, and PR-M, analyzed the data. EG-G and CW wrote the manuscript. All authors read and approved the final manuscript.

## Conflict of Interest

The authors declare that the research was conducted in the absence of any commercial or financial relationships that could be construed as a potential conflict of interest.
